# Supplementary material for: Feeding Interventions for Infants with Growth Failure in the First Six Months of Life: A Systematic Review
Source: Nutrients. 2020 Jul 9;12(7):2044. doi: 10.3390/nu12072044 (PMC7400880; doi:10.3390/nu12072044)
Supplement: Supplementary file 1 [file nutrients-12-02044-s001.pdf]

Table S1. Information on the declaration of financial support and conflict of interest

Author (year)	Population Characteristics	Intervention	Funding	Conflict of Interest
**Cup feeding interventions (n=2)**				
Abouelfettoh (2008)	Preterm (LBW)<br>[GA: 35.13wk, Bwt: 2150g]<br>Recruitment: NICU	**Cup feeding**<br>IG: Only cup feeding<br>CG: Only bottle feeding	Not mentioned in the paper	None declared
Yilmaz (2014)	Preterm (VLBW)<br>[GA: 32-35wk, Bwt: 1543g]<br>Recruitment: NICU	**Cup feeding**<br>G1: Cup feeding<br>G2: Bottle feeding	Authors received no financial support	None declared
**Formula Fortification/Supplementation interventions (31)**				
Abrams (2014)	Preterm (VLBW)<br>[Bwt: <1,250g]<br>Recruitment: NICU	**Cow milk**<br>G1: HM [HM (mother's own/donor milk) + HM based fortifier]<br>G2: CM [cow milk formula + cow milk-based fortifier]	Prolacta Bioscience	None declared
Alan (2013)	Preterm (VLBW)<br>[GA: ≤32wk, Bwt: ≤1500g]<br>Recruitment: NICU	**Protein supplementation**<br><u>When mothers expressed first milk</u><br>IG: HM with extra protein supplementation<br>CG: HM with a standard fortification	Authors received no financial support	None declared
Arslanoglu (2006)	Preterm (LBW, VLBW, ELBW)<br>[GA: 26-34wk, Bwt: 600-1750g]<br>Recruitment: Hospital	**Protein supplementation**<br><u>When volume reached 150ml/kg/d</u><br>G1: HMF + additional protein<br>G2: HM with HMF in the standard amount	Authors received no financial support	Not mentioned in the paper
Florendo (2009)	Preterm (VLBW)<br>[GA: ≤32wk, Bwt: 1200g]<br>Recruitment: New born medical centre	**Protein supplementation**<br>IG: Partially hydrolysed whey protein<br>CG: Non-hydrolysed whey casein preterm infant formula	Nestec Ltd, Vevey, Switzerland	Not mentioned in the paper
Kim (2015)	Preterm (VLBW)<br>[GA: ≤33wk, Bwt: 1174g]	**Protein supplementation**<br><u>When volume reached 100ml/kg/d</u>	Abbott Nutrition	None declared

Author (year)	Population Characteristics	Intervention	Funding	Conflict of Interest
	Recruitment: NICU	IG: Conc. HMF containing liquid extensively hydrolysed protein<br>CG: Powdered intake protein HMF		
Erasmus (2002)	Preterm (VLBW)<br>[GA: 26-34wk, Bwt: 1407g]<br>Recruitment: NICU	**Lactase fortification**<br>From birth (day 1) to 36 wk or discharged<br>IG: Fortified HM or preterm formula treated with lactaid drops (Lactase)<br>CG: Untreated fortified HM or preterm formula	Authors received no financial support	Not mentioned in the paper
Gathwala (2007)	Term SGA (LBW)<br>[GA: 40wk, Bwt: 2000g]<br>Recruitment: Hospital	**Lactase fortification**<br>When volume reached 100ml/kg/d<br>IG: HM fortified with Lactodex-HMF<br>CG: Only BM	Authors received no financial support	Not mentioned in the paper
Berseth (2004)	Preterm (VLBW)<br>[GA: ≤33wk, Bwt: 1180g]<br>Recruitment: Hospital	**Iron fortification**<br>When volume reached 100ml/kg/d<br>G1: HMF (iron fortified)<br>G2: HMF (standard)	Mead Johnson Nutritionals	Berseth, Stolz, Harris and Hansen are emloyees of Mead Johnson Nutritionals
Willeitner (2017)	Preterm (VLBW, ELBW)<br>[GA: 29wk, Bwt: 500-1499g]<br>Recruitment: NICU	**Iron fortification**<br>Birth- day 3<br>IG: HM fortification (Conc. Preterm Formula 30- Similac Special Care 30 with iron)<br>CG: Standard Powdered HMF (Similac HMF)	Not mentioned in the paper	Not mentioned in the paper
Clarke (2007)	Faltering growth<br>[GA: 2-31wk]<br><3rd centile for weight and height for age; weight gain <50% of expected<br>Recruitment: Children's Hospital	**Nutrient fortification**<br>G1: Nutrient-dense formula<br>G2: Energy-supplemented formula	Nutricia Clinical Care	None declared
Morlacchi (2016)	Preterm (VLBW)<br>[GA: <32wk, Bwt: 1255g]	**Nutrient fortification**<br>First day	Authors received no financial support	Not mentioned in the paper

Author (year)	Population Characteristics	Intervention	Funding	Conflict of Interest
	Recruitment: NICU	G1: Macronutrient fortification<br>G2: Standardized fortification (Aptamil BMF, FM85)		
Worrell (2002)	Preterm (VLBW)<br>[GA: 27±3wk, Bwt: 925g]<br>Recruitment: NICU	**Nutrient fortification**<br>G1: Transitional formula (TF-higher amounts of protein, Ca, P, and several trace minerals and vitamins)<br>G2: Standard formula	Not mentioned in the paper	Not mentioned in the paper
Cristofalo (2013)	Preterm (ELBW)<br>[GA: <27wk, Bwt: 989g]<br>Recruitment: NICU	**Bovine milk**<br>G1: Exclusive appropriately fortified HM<br>G2: Bovine milk-based preterm formula<br>1-4d after birth and continued at 10-20 ml/kg/d as tolerated for up to 5 days	Prolacta Bioscience	Not mentioned in the paper
Hair (2014)	Preterm (ELBW)<br>[GA: 28wk, Bwt: 970g]<br>Recruitment: NICU	**Cream supplementation**<br>IG: HM derived cream supplement<br>CG: Mothers own milk or donor's HM derived fortifier	US Dept. of Agriculture/Agriculture Research Service, National Centre for Research resources General Clinical research for Children, Prolacta Bioscience	None declared
Shah (2016)	Preterm (VLBW)<br>[GA: 27wk, Bwt: <1500g]<br>Recruitment: NICU	**Early and delayed fortification**<br>G1: Early fortification (20ml/kg/d of HM feeds)<br>G2: Delayed fortification (100ml/kg/d of HM feeds)	Not mentioned in the paper	Not mentioned in the paper
Taheri (2016)	Preterm (VLBW)<br>[GA: 28-34wk, Bwt: 1294g]<br>Recruitment: NICU	**Early and delayed fortification**<br>G1: Early fortification (1st feeding)<br>G2: Late fortification (BF volume reached 75ml/kg/d)	Not mentioned in the paper	Not mentioned in the paper
Tillman (2012)	Preterm (VLBW)<br>[GA: <31wk, Bwt: 1123g]<br>Recruitment: Neonatal database (NICU)	**Early and delayed fortification**<br>Fortification with Enfamil, powdered HM fortifier<br>G1: Early BM fortification (1st feed)	Not mentioned in the paper	Not mentioned in the paper

Author (year)	Population Characteristics	Intervention	Funding	Conflict of Interest
		G2: Delayed fortification (when volume reached 50-80ml/kg/d)		
Bhat (2001)	Preterm (VLBW)<br>[GA: 26-34wk, Bwt: 1242g]<br>Recruitment: Special care baby unit	**Human milk fortification**<br><u>When clinical conditions permitted</u><br>IG: Fortified HM<br>CG: HM only<br>Dose: 1g of fortifier added to 100ml of milk on day 1, and gradually increased to 4g added to 100ml on 3rd/4th day	Not mentioned in the paper	Not mentioned in the paper
Morlacchi (2018)	Preterm (VLBW)<br>[GA: <32wk, Bwt: <1500g]<br>Recruitment: NICU	**Human milk fortification and formula**<br>G1: Fortified HM<br>G2: Preterm formula	Authors received no financial support	None declared
Kim (2017)	Preterm (ELBW)<br>[GA: <32 wk, Bwt: 1087g]<br>Recruitment: NICU	**Human milk and formula**<br>G1: Donor human milk<br>G2: Preterm formula<br><u>Infants fed G1&G2 before achieving an enteral intake volume of 130 ml/kg/d</u>	Not mentioned in the paper	None declared
Lok (2017)	Preterm (LBW, VLBW)<br>[GA: <37wk, Bwt: <2200g, VLBW: <1500g,<br>LBW: ≥1500g and <2200g]<br>Recruitment: NICU	**Human milk and formula**<br><u>1-4d after birth and continued at 10-20 ml/kg/d as tolerated for up to 5 days</u><br>Category 1: LBW, Category 2: VLBW; Both the groups further divided into-<br>G1: Any BM (human/donor)<br>G2: No BM (infant formula)	Small Project Fund of Univ. Of Hongkong	None declared
Manea (2016)	Preterm (ELBW)<br>[GA: 25-33wk, Bwt: <1,000g]<br>Recruitment: Children hospital	**Human milk and formula**<br><u>Once enteral nutrition (24-48 hrs of life) started until initiation of bottle feeding</u><br>G1: Human BM<br>G2: Formula	Not mentioned in the paper	Not mentioned in the paper
Morley (2000)	Preterm (LBW)<br>[GA: ≤31wk, Bwt: <1850g]<br>Recruitment: Neonatal unit	**Human milk and formula**<br><u>Fed until they reach wt. of 2000g or discharged from NICU</u>	Farley Health Products (division of HJ Heinz Company Ltd.)	Not mentioned in the paper

Author (year)	Population Characteristics	Intervention	Funding	Conflict of Interest
	and breast milk bank centre/ without BM banks	Category 1: As sole diet<br>Category 2: As supplement to HM<br>G1: Banked donor milk<br>G2: Preterm formula		
O'connor (2016)	Preterm (ELBW)<br>[GA: 27.5wk, Bwt: 995g]<br>Recruitment: NICU	**Human milk and formula**<br>Initiated after birth and advanced at a rate of 10-25 ml/kg/d up to 160ml/kg/d<br>G1: Donor milk<br>G2: Preterm formula	Not mentioned in the paper	Not mentioned in the paper
Moya (2012)	Preterm (ELBW)<br>[GA: ≤30wk, Bwt: 1000g]<br>Recruitment: NICU	**Liquid and powdered fortification**<br>From birth to 28 days<br>G1: Liquid HMF<br>G2: Powdered HMF	Mead Johnson Nutrition	Not mentioned in the paper
Kanmaz (2012)	Preterm (ELBW)<br>[GA: 28wk, Bwt: 1092g]<br>Recruitment: NICU	**Different levels of fortification**<br>When full feedings were achieved<br>G1: Standard fortification [1.2g HMF+30ml HM]<br>G2: Moderate fortification [1.2g HMF+25ml HM]<br>G3: Aggressive fortification [1.2g HMF+20ml HM]	Authors received no financial support	None declared
Porcelli (1999)	Preterm (VLBW, ELBW)<br>[GA: 25-32wk, Bwt: 600-1500g]<br>Recruitment: NICU	**Different fortifier**<br>G1: Test HMF (1 g of protein/100 ml of supplemented milk, 85% glucose polymers, 15% lactose, and calcium, phosphorus, sodium, copper)<br>G2: Reference HMF (60% whey protein and 40% casein, 75% glucose polymers, 25% lactose and calcium, phosphorus, sodium, and copper)	Wyeth Nutritionals International, Philadelphia, USA	Not mentioned in the paper
Kumar	Preterm (ELBW)	**Different formula**	Not mentioned in the paper	None declared

Author (year)	Population Characteristics	Intervention	Funding	Conflict of Interest
(2017)	[GA: 27wk, Bwt: 993g]<br>Recruitment: NICU	G1: Similac liquid human milk fortifier (Similac Human Milk Fortifier Hydrolyzed Protein Conc. Liquid)<br>G2: Enfamil liquid human milk fortifier (Enfamil human milk fortifier acidified liquid)		
Amesz (2010)	Preterm (VLBW)<br>[GA: ≤32wk, Bwt: 1338g]<br>Recruitment: Neonatal unit	**Different formulas**<br><u>Until term CA</u><br>G1: Post discharge formula<br>G2: Term formula<br>G3: HM fortified formula	Friesland Food, Leeuwarden, the Netherlands	None declared
Lucas (1992)	Preterm (VLBW)<br>[GA: 31wk, Bwt: 1475g]<br>Recruitment: NICU	**Different formula**<br>G1: Follow-on preterm formula (FPF- Farley's Premcare)<br>G2: Standard term formula (STF-Farley's Oster Milk)	Farley Health Products	Not mentioned in the paper
Flaherman (2013)	Term infants<br>[>37 wk who lost ≥5% Bwt before 36 hrs of age]<br>Recruitment: Children hospital	**Continued EBF and early limited formula**<br><u>Who lost ≥5% of birth weight before 36 hrs</u><br>IG: Early limited formula (ELF 10 ml using feeding syringe)<br>CG: Continued EBF	National Institutes of Health (NIH)	One of the author served as a paid consultant for the following companies: Abbott Nutrition (Abbott Park, IL), Mead-Johnson (Evansville, IN), Nestle SA (Vevey, Switzerland), and Pfizer Consumer Products (Madison, NJ)
**Enteral feed interventions (n=8)**				
Akintorin (1997)	Preterm (VLBW, ELBW)<br>[GA: 28wk, Bwt: 700-1250g]	**CNG and IBG feeds**	Not mentioned in the paper	Not mentioned in the paper

Author (year)	Population Characteristics	Intervention	Funding	Conflict of Interest
	Category 1: 700-1000g<br>Category 2: 1001-1250g<br>Recruitment: NICU	Parenteral nutrition started on days 2 to 3 and continued until each infant tolerated full enteral feedings<br>G1: CNG vs IBG<br>G2: CNG vs IBG		
Mosqueda (2008)	Preterm (ELBW)<br>[GA: 26wk, Bwt:760g]<br>Recruitment: NICU	**Intravenous and nasogastric feeds**<br>G1: Intravenous alimentation alone (NPO-None per orem)<br>G2: Small boluses of nasogastric feedings (Minimal enteral nutrition)	Not mentioned in the paper	Not mentioned in the paper
Kliethermes (1999)	Preterm (LBW)<br>[GA: ≤32wk, Bwt: 1685g]<br>Recruitment: Regional perinatal centre	**Nasogastric and bottle feeds**<br>G1: Nasogastric tube<br>G2: Bottle feeding	Not mentioned in the paper	Not mentioned in the paper
Bora (2017)	Preterm (VLBW)<br>[GA: 35wk, Bwt: 1357g]<br>Recruitment: NICU	**Complete and minimal feeds**<br>G1: Complete enteral feed (CEF) with EBM<br>G2: Minimal enteral feed (MEF) with IVF (trophic feeds 20 ml/kg of EBM and 60ml/kg 10% Dextrose by IV route)	Not mentioned in the paper	Not mentioned in the paper
Colaizy (2012)	Preterm (ELBW)<br>[GA: 27wk, Bwt: 889g]<br>Recruitment: NICU	**Different levels of feeds**<br>Total enteral intake as HM, donor milk, Mixed HM/DM<br>G1: <25%, G2: 25-50%, G3: 50-75%<br>G4: >75%	NIH K23HD057232	None declared
Thomas (2012)	Preterm (VLBW)<br>[GA: 31.7wk, Bwt: 1220g]<br>Recruitment: NICU	**High and standard volume feeds**<br>G1: High volume feeds (300ml/kg/d of EBM)<br>G2: Standard volume feeds (200ml/kg/d of EBM)	Not mentioned in the paper	Not mentioned in the paper
Salas (2018)	Preterm (ELBW)<br>[GA: 22-28wk, Bwt: 833g]<br>Recruitment: NICU	**Early and delayed feeding**<br>G1: Early progressive feeding without trophic feeding	Gerber Foundation WAC and NIH (Grants U10 HD 34216 and ULTTR001417)	None declared

Author (year)	Population Characteristics	Intervention	Funding	Conflict of Interest
		G2: Delayed progressive feeding after 4d course of trophic feeding		
Zecca (2014)	Preterm (LBW)<br>[GA: 32-36wk, Bwt: >1499g]<br>Recruitment: NICU	**Proactive and standard feeds**<br>G1: Proactive Feeding Regimen (1st d of life-100ml/kg/d of HM, day 2-130ml/kg/d, day 3-165ml/kg/d, day 4-discharge 200ml/kg/d)<br>G2: Standard Feeding Regimen (1st d of life-60ml/kg/d of HM and gradually increased to170ml/kg/d by day 9)	Not mentioned in the paper	None declared
**Other interventions (n=6)**				
Aly (2017)	Preterm (VLBW)<br>[GA: ≤34wk, Bwt: 1300g]<br>Recruitment: NICU	**Bee honey**<br>G1: 5g, G2: 10g, G3: 15g<br>G4: 0g (control)	Not mentioned in the paper	None declared
Heon (2016)	Mothers of EP infants<br>Recruitment: NICU	**Electric breast pump**<br>IG: Standard care + double electric breast pump + BM expression education and support intervention<br>CG: Education and support	AWHONN, Canadian Institute of Health Research, Groupe de recherche interuniversitaire en interventions en sciences infirmieres du Quebec (GRIISIQ), Medela Canada	None declared
Slusher (2007)	Mothers of preterm<br>[GA: 31wk]<br>Recruitment: Teaching and mission hospital	**Electric breast pump**<br>G1: Electric breast pump<br>G2: Non-electric pedal Pump<br>G3: Hand expression	Not mentioned in the paper	Not mentioned in the paper
Kumar (2010)	Preterm (VLBW)<br>[GA: ≥32wk, Bwt: >1250-≤1600g]<br>Recruitment: Tertiary level Neonatal unit	**Nasogastric and spoon feeds**<br><u>Trial 1</u><br>G1: NG feeding in hospital<br>G2: Spoon feeding in hospital<br><u>Trial 2</u><br>G1: Spoon feeding in hospital<br>G2: Spoon feeding at home	Not mentioned in the paper	Not mentioned in the paper

**Author (year)**	**Population Characteristics**	**Intervention**	**Funding**	**Conflict of Interest**
Lau (2012)	Preterm (VLBW)<br>[GA: 28wk, Bwt: 1103g]<br>Recruitment: NICU	**Suckling and swallowing**<br>IG1: Non-nutritive sucking exercise (pacifier use)<br>IG2: Swallowing exercise (placing a milk/formula bolus through syringe)<br>CG: Standard care	Authors received no financial support	None declared
Serrao (2018)	Mothers of preterm<br>[GA: 27-32wk]<br>Recruitment: Previously registered trial	**Galactagogue**<br>From 3rd -28th d after delivery<br>G1: Silymarin-phosphatidylserine and galega (a daily single dose of 5 g of Piu`latte Plus MILTE)<br>G2: Placebo (a daily single dose of 5 g of lactose)	Authors received no financial support	None declared
